# Brain Networks Reorganization During Maturation and Healthy Aging-Emphases for Resilience

**DOI:** 10.3389/fpsyt.2018.00601

**Published:** 2018-11-21

**Authors:** Gabriel Gonzalez-Escamilla, Muthuraman Muthuraman, Venkata C. Chirumamilla, Johannes Vogt, Sergiu Groppa

**Affiliations:** ^1^Department of Neurology, University Medical Center, Johannes Gutenberg University Mainz, Mainz, Germany; ^2^Institute for Microscopic Anatomy and Neurobiology, University Medical Center, Johannes Gutenberg University Mainz, Mainz, Germany

**Keywords:** resilience, lifespan, brain networks, brain reorganization, health maintenance

## Abstract

Maturation and aging are important life periods that are linked to drastic brain reorganization processes which are essential for mental health. However, the development of generalized theories for delimiting physiological and pathological brain remodeling through life periods linked to healthy states and resilience on one side or mental dysfunction on the other remains a challenge. Furthermore, important processes of preservation and compensation of brain function occur continuously in the cerebral brain networks and drive physiological responses to life events. Here, we review research on brain reorganization processes across the lifespan, demonstrating brain circuits remodeling at the structural and functional level that support mental health and are parallelized by physiological trajectories during maturation and healthy aging. We show evidence that aberrations leading to mental disorders result from the specific alterations of cerebral networks and their pathological dynamics leading to distinct excitability patterns. We discuss how these series of large-scale responses of brain circuits can be viewed as protective or malfunctioning mechanisms for the maintenance of mental health and resilience.

## Introduction

Aging is related to alterations of cognitive functioning accompanied by structural and functional brain reorganization (1, 2). Maintained cognitive function late in life is generally achieved by the integrated communication of specific brain regions (3, 4). The functional and structural reorganization of brain circuits occur continuously during the lifespan and play an essential role for preserving brain health (5–7). Hence, abnormal cognitive function may build upon specific alterations of brain networks and their dynamic responses to life events or physiological processes during maturation or aging (8–10). An exact understanding of structural and functional longitudinal properties and a precise characterization of the tissue properties are crucial for modeling the long-term processes and to distinguish between healthy and disease-specific alterations. Hence, modeling interregional connectivity and specific reorganization of cerebral networks topology is likely to promote our understanding of underlying mechanisms of mental health and resilience to life events (11).

**Figure 1:**
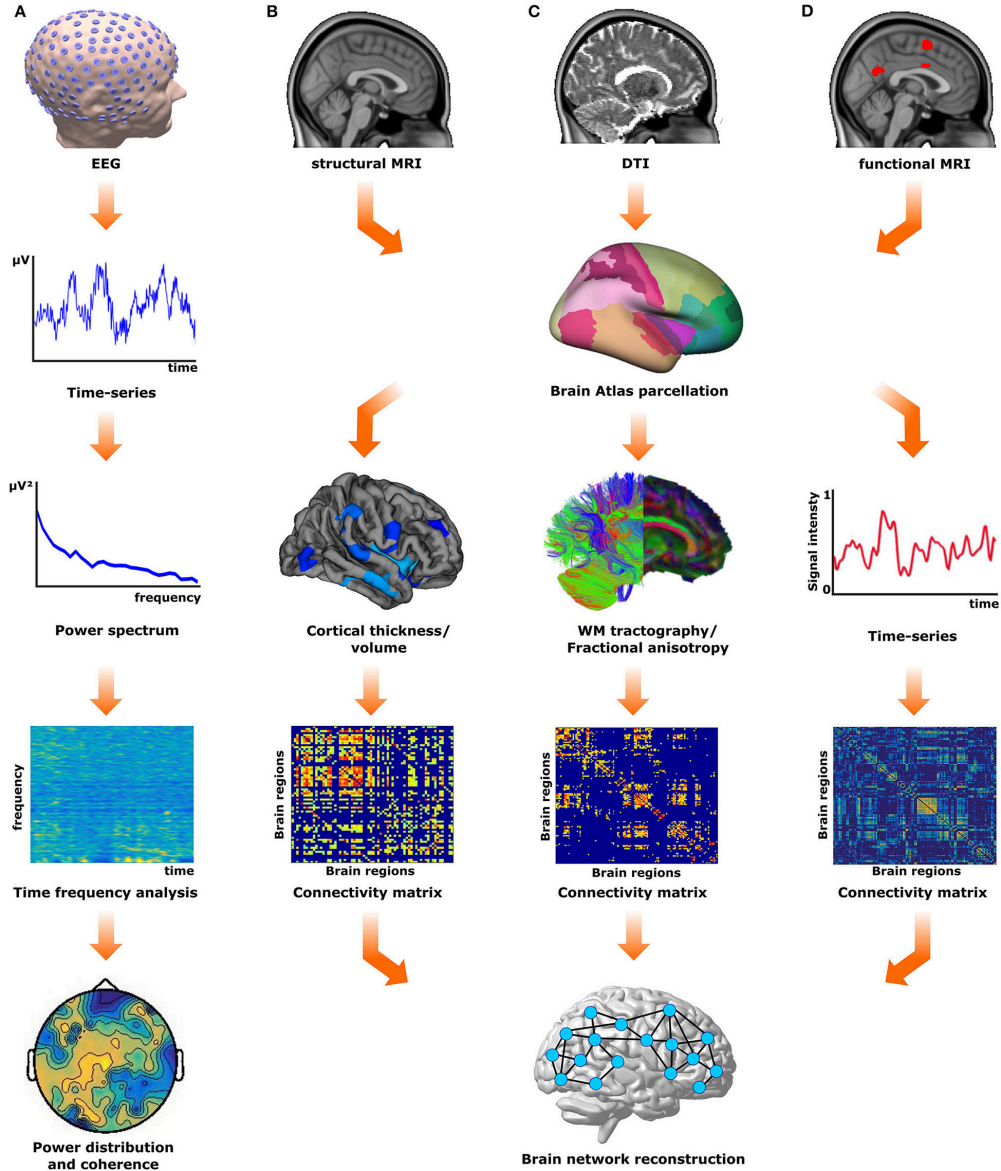
Overview of network reconstruction methods. **(A)** The electrical activity of the brain is recorded using electroencephalography (EEG). These recordings (EEG time-series) are analyzed using time-frequency analysis approaches to investigate the spatiotemporal distribution of the frequency power. **(B)** From structural (T1) magnetic resonance images (MRI) morphological measures (cortical thickness/volume) for different brain regions can be extracted according to a predefined atlas. These measures are used to obtain a structural covariance matrix, from which the structural gray matter network is reconstructed. **(C)** The diffusion tensor images (DTI) are used to derive white matter tracts, from either probabilistic or deterministic tractography algorithms, or fractional anisotropy maps. These measures are used to obtain a connectivity matrix according to the brain atlas of choice, and subsequently, the structural white matter network is reconstructed. **(D)** The functional MRI (fMRI) time series from different brain regions obtained can also be used to generate the functional connectivity matrix and subsequently to reconstruct the functional brain network.

Functional connectivity patterns can be obtained from the temporal correlations of spontaneous neurophysiological signal fluctuations between brain regions either by electroencephalography (EEG) or functional magnetic resonance imaging (fMRI), and then analyzed within a graph-theoretical framework (7, 12, 13). On the other hand, the structural network connectivity can be accessed by pure structural MRI measures like cortical thickness and volume or by white matter fiber tracts obtained through tractography between predefined regions of interest (6, 14, 15) as shown in Figure 1. These network fingerprints are predictive measures of disease-related clinical symptoms (6, 7) or therapy outcomes (16).

In this review, we present existing evidence for a profound understanding of lifespan-related reorganization processes that can be related to protective mechanisms that help our brain to cope with age-related situations and reduce the burden for brain alterations linked to mental disorders. Unraveling complementary functional and structural fingerprints should give important insight on inter-individual courses. On the basis of our recent results on the importance of the cerebral networks in disease outcome (6, 13, 16–19), we discuss the impact of the structural gray matter tissue integrity and reorganization of normal appearing white matter and evolving functional adaptations for clinical phenotypes. Non-invasive structural and fMRI characterization of the neuronal circuits will be discussed from a longitudinal perspective.

We hypothesize that (i) cerebral networks in resilient subjects with preserved mental health despite traumatic events are characterized by a reorganization of the gray and white matter compartments with a strengthening of distinct regional connectivity patterns and preserved structural integrity in the key anatomical regions (prefrontal cortex, hippocampus and corpus callosum); (ii) these brain circuits remodeling processes are partially mirrored in age-related reorganization during maturation or healthy aging; (iii) neurocognitive and clinical impairment of mental health is associated with exhaustion of network compensation, which manifests in divergent lifespan patterns of network reorganization or a breakdown of functional responses; (iv) structural and functional lateralization patterns together with inter-hemispheric connections and the connectivity fingerprints in the above mentioned networks together with their functional interactions have a large impact on network compensation and thus inter-individual mental status. The overall aim of this work is to identify distinct network connectivity and integrity patterns reflecting compensation processes for global network functioning. We focus first on maturation processes and healthy aging and drive parallels to mechanisms of resilience behavior.

## Age-related brain remodeling and excitability dynamics during maturation

Accurate synaptic transmission is a fundamental requirement for normal brain function (20); signaling alterations at the excitatory synapse leading to cortical hyperexcitability have been related to psychiatric disorders such as schizophrenia (21–24). However, to what extent alterations in brain maturation leading to cortical miswiring and subsequent cortical hyperexcitability contribute to psychiatric disorders has not been fully elucidated. During brain development and maturation, neuronal activity is an important regulator of cortical connectivity. Experimental data suggests that disrupted neuronal activity during circuit maturation results in a failure of the refining of the circuit, inducing miswiring and increased network hyperexcitability due to alterations of the postsynaptic compartment (25, 26). In line with this, recent data shows that during juvenile brain development neuronal activity is needed for the proper formation of interhemispheric connections, while inhibition of neuronal activity resulted in decreased neuronal connectivity (27). In outgrowing axons, increased neuronal activity and proper connectivity was suggested to depend on axonal Ca^2+^-signaling leading to activation of the CaMKK/CaMKI alpha cascade, thereby supporting axonal outgrowth (28). However, although neuronal activity was shown to be also important for subcortical-cortical projections, like the thalamo-cortical fibers (29), recent data suggests that pathological increased cortical excitability during juvenile brain development affects the formation of cortical connections leading to decreased cortical connectivity (30). Thus, neuronal activity during brain maturation has to be balanced: too low or too high neuronal activity levels are associated with cortical miswiring and cortical network hyperexcitability and may lead to psychiatric disorders at further adult ages. Indeed, alteration of cortical excitation/inhibition (E/I) balance and subsequent alteration of cortico-cortical modulation was shown to lead to increased cortical gamma oscillations (31, 32). Moreover, recent human data has shown that schizophrenia patients display increased spontaneous gamma activity during auditory steady-state stimulation reflecting a disrupted E/I balance (33).

A second critical period of cortical restructuring, which is present in distinct and interrelated connectivity development and cortical regions activity shaping, occurs during adolescence (34). Twin studies suggest that these cortical growth trajectories are determined by different sets of genes, which are active in interconnected brain subregions (35). Further studies revealed that regional alterations in the gray matter properties occurred in specific brain networks, which are relevant for the development of psychiatric disorders. Reorganization of these brain networks in adolescence is suggested to result in a particular vulnerability for psychiatric disorders (36, 37). Indeed, analyses in patients with childhood-onset schizophrenia have identified an abnormal pattern of cortical growth in the cingulo-fronto-temporal area of these patients, and suggest a specific impact of genetic systems in these neuroanatomical modules affecting their connectivity (38). Interestingly, network-specific alterations, which increase vulnerability to brain disorders, are not restricted to developmental periods, but have also been found to be present in different neurodegenerative disorders (39). These data suggest that developmental disturbances during adolescence, leading to increased vulnerability to psychiatric and neurodegenerative disorders rely on network-driven alterations of specific brain networks.

The idea that altered neuronal connectivity during brain maturation may lead to psychiatric disorders is supported by longitudinal studies focusing on delayed development of brain connectivity. Adolescents with childhood-onset of schizophrenia, as well as their clinically unaffected siblings, showed reduced structural integrity and connectivity deficits in the left occipito-temporal areas (40). Although the maturation deficits of cortical connectivity have been shown to become normal with age in patients' siblings (41), this is not the case for the adolescents with childhood-onset schizophrenia (42). These findings support the hypothesis that maturation of cortical connectivity is an important factor for resilience to psychiatric disorders, in which alterations in cortical connectivity at certain life periods, as present in adolescents, may affect resilience toward psychiatric disorders. This is further supported by a recent study which correlated the microarchitecture of corpus callosum to the ability of individuals, who were exposed to high stress, to resist mental disorders (43). Here, young adolescents (mean age: 14.4 ± 1.31 years) with a high resilience to psychiatric disorders displayed higher fractional anisotropy (FA) values in the anterior corpus callosum when compared to adolescents at-risk for mental disorders or with controls (43).

In addition, findings showing altered morphological connectivity following abnormal adolescent brain maturation and associated with cortical hyperexcitability have taken a central role for the current view on the development of psychiatric disorders (24). This is in line with findings that early cortical hyperexcitability has a deleterious effect on brain development leading to sequelae later in life. For instance, Dube and co-workers (44) have shown that stress during sensitive early life periods led to cortical hyperexcitability at later life periods, where 57% of the individuals with early life stress developed epileptic seizures (44). However, and despite the immediate drastic effects like epileptiform discharges (5), continued and sustained cortical hyperexcitablility may lead to psychiatric disorders, as described in animal models for autism phenotypes (45), or may contribute to the pathological cascade of events that contribute to the development of Alzheimer's disease (46). Interestingly, not only generalized cortical excitability, but hyperexcitability of specific brain regions was shown be involved in fear reactions and reduced extinction suggesting an important link between neuronal excitability of specific brain regions like the dentate gyrus in the pathology of post-traumatic stress disorder. In sum, proper maturation from the synaptic level up to the cortical circuit level assuring correct neurotransmission and cortical connectivity is a prerequisite for proper resilient behavior at adult ages, while alterations in E/I balance and in cortico-cortical connectivity may lead to psychiatric disorders.

## Brain reorganization and network compensation during aging

From early life through adulthood the brain is constantly changing; later in life the physiological aging process (i.e., free from neurodegeneration) is associated with modification of intrinsic neuronal excitability, together with functional and structural connectivity reorganization. These processes are typically associated with preservation or rather decline in performance across several cognitive domains. In elderly people, preserved function is thought to be underpinned by compensatory mechanisms (47), in which proximal or distal brain regions to those that decline over time because of natural aging, are recruited to maintain function (48).

From the functional perspective, age-associated adaptations of intrinsic neuronal excitability have been related with changes in cellular micro-architecture (i.e., membrane ion channels, receptors and vesicle fusion processes) and its molecular signaling (49, 50). Although the mechanisms of action are not yet fully understood, numerous evidence has shown involvement of ion-gated channels (e.g., voltage-gated Ca^2+^ channels and mechano-gated K^+^ channels) in mediating the loss of plasticity in neurons, making neurons more susceptible to deleterious processes such as oxidative stress (51). As an example, previous studies have shown increased loss of dopamine synthesis with healthy aging in the striatal system (52), which is particularly vulnerable to oxidative stress (53, 54). At this stage in life, the rate of dopamine loss is more prominent than the regional loss of gray matter tissue (52) and is related to cognitive performance (55), hence suggesting that this transition could be initiated by changes in the ion channels. However, further research is still needed in order to unveil the importance and to better characterize the impact of molecular changes related to the wide-spread functional and structural brain changes associated with healthy aging.

At the macro level, extra-cephalic electrophysiological recordings have repeatedly evidenced age-related alterations of oscillatory activity across distributed portions of the cortex. Since a loss of approximately 10% of all neocortical neurons over the lifespan occurs (56), alterations of local activity can be explained by an impaired synchronization of neuronal activity in specific frequency ranges. One possible cause of impaired synchronous firing activity can be an alteration of the cortico-cortical neuromodularity mechanisms or imbalances in the subcortico-cortical circuits, directly impacting spontaneous neuronal firing rates and changing the E/I balance. This appears to be the case for high frequency bands, for instance beta (13–30 Hz) and gamma (30–45 Hz) bands, in which increased power has been reported with increasing age (57), whereas decreases in lower alpha (8–10.5 Hz) and a slowing of peak alpha frequency appear with aging (58). Here, age-related interhemispheric asymmetry in power has been related to an increased excitability within the sucortico-cortical circuits (57), which is interpreted as a compensatory mechanism. However, a less consistent scenario emerges for frequencies in the slow wave range, delta (1–4 Hz) and theta (4–7.5 Hz) bands, in which both increases and decreases have been reported (59–62). Notably, apart from increased functional activity (47, 63), reduction of brain lateralization has been described as an important age-related mechanism for compensatory functions (64). However, this view considers only detrimental aspects of aging toward pathology and ignores a causal dynamical relationship with neural adaptation (i.e., plasticity) to life events (e.g., education, intellectual engagement and daily activities) during maturation.

On the structural part, although annual decreases on the order of 0.2–0.5% are well documented (65), MRI-derived morphometric measures (gray matter volume and thickness) have demonstrated heterogeneous effects of aging, in which despite occurrence of disseminated atrophy across the whole brain, changes vary from region to region and tissue type, particularly over the cortical mantle (65, 66). Of all cortical regions, the frontal and parietal cortices appear the most susceptible to age-related changes (65, 67–70), with accumulating evidence showing increased involvement of the temporal regions (65). At the subcortical level, the hippocampus, caudate nucleus and cerebellum are the most age-susceptible regions (67), whereas involvement of regions belonging to the limbic system appear only limited (71–74). How these changes participate or guide aging processes is still unknown. For instance, reduction of the integrity in the prefrontal cortex is related with functional hyper-activation of the same region during task performance, suggesting the existence of compensatory mechanisms that support the maintenance of cognitive function (1, 75). Anatomical brain lateralization also seems to be nontrivial during aging, since for example, a trend for faster gray matter loss of the left prefrontal cortex relative to the right one has been described (76). Although this finding has not been consistently reported (77), individuals with smaller left than right hemispheric structural integrity are more likely to report cognitive deficits (78). Furthermore, converging evidence exists that an aging-related asymmetric loss of integrity in the parietal and temporal cortices is associated with cognitive functioning (79).

Examination of the white matter tissue has pointed to reduced microstructural integrity in the fiber tracts of the frontal and parietal lobes, as well as in the corpus callosum in elderly persons (80–82). White matter alterations are associated with decrements in cognitive performance, speed of processing, memory and executive functions (82). Moreover, age-related metabolic decreases in the middle and superior temporal cortex, albeit less pronounced than in frontal regions, have been related to white matter disturbances in the long fronto-temporo-occipital association pathways (83). Although these changes are likely to be explained by changes in myelin content (84), due to its influence on signal conduction (85), the effects of age on myelin are complex; even though some reduction in myelin sheaths is observed with age, the myelin is continuously produced throughout life, but perhaps in an uncontrolled or dysfunctional manner (86). Recent advances in MRI methods have successfully ascertained the *in-vivo* assessment of myelin content, showing a negative correlation with aging in the white matter (87, 88) and also to a lesser extent the gray matter (89). Notably, separation of myelin effects from cerebrovascular alterations is not easy using MRI approaches (90), because focal age-related anatomical changes in the white matter, e.g., white matter lesions, can also result from changes in blood pressure (91).

## Impact of divergent patterns of network reorganization for resilience and sustained mental health

Detection of altered brain circuits in the comparison between healthy subjects and patients only partially captures the neurobiological complexity of reorganization processes associated with maturation and aging and their influence on the development of pathological trajectories (92). The modern, network view of the human brain envisions circuits that are not only shaped by interactions (connections) between their constituent elements (brain regions), but also by their complex topological organization and temporal dynamics These factors mainly determine the differentiation between physiological vs. patholological processes and hold the key to describing reorganization associated with resilience and sustained mental health (11). Hence, the efficient organization of the brain networks results from delicate balancing of the opposing requirements for information integration and segregation, allowing effective complex cognitive and perceptual functions essential for mental health (93, 94).

Functional connectivity during resting state (i.e., individuals are relaxed and awake, but not engaged in task-directed cognition) has been increasingly applied to investigate the reorganization of the brain across the lifespan, showing that the network topology has a tendency to become randomly organized with increasing age, losing its efficient organization. Such dynamics seem to be good predictors of the individual transition from young to middle age (95), where the information flows from and to the frontal and parietal regions have a primordial role for physiological preservation of mental function.

Interestingly, despite the amount of research showing that chronic exposure to high levels of stress is associated with increased susceptibility to anxiety and other mental disorders (96–98), there is compelling evidence that more graded exposure to stress might reduce such vulnerabilities and promote resilience (99) or may even positively influence several cognitive domains such as memory functions influencing cerebral networks encompassing the hippocampus and amygdala (100). Of note, the stress responses mediated by the amygdala are regulated by the medial prefrontal cortex and their coupling (101–103); thus, stress exposure impairs prefrontal cortex-mediated cognitive functions and switches the control of stress behavior and emotion to interconnected brain circuits (104). These results support the hypothesis of compensation (105), establishing that the recruitment of secondary networks is a mediator of the relationship between structural brain damage and memory or targeted attacks (13). Hence, failure in the compensatory processes related to stress could reduce the ability of the brain circuits to compensate insults, increasing the rate at which functioning is impaired by new challenges (106). Indeed, sustained activation of the circuits involved in coping with stress situations could pass from being an adaptive or compensatory response, to lead to impairments in learning, memory, and the ability to regulate future stress responses (107, 108) and increase the vulnerability to a range of mental disorders over a lifetime (109). On the contrary, forms of early enrichment could induce an accurate heterotypic adjustment at molecular, synaptic and brain circuits levels that could strengthen resilience behavior (99, 110).

In some clinical conditions, such as depression or post-traumatic stress disorder (PTSD), an impaired structural network with abnormal hippocampal integrity and diminished function was described and considered as disease landmarks (111, 112). Smaller hippocampal volumes have been as well attested in women with major depression and related to experiences of childhood trauma, while depressed individuals without similar trauma events had hippocampal volumes similar to healthy controls (113). Moreover, the unexposed twins of PTSD patients show a similar degree of hippocampal decrease, but without clinical implications (114). Hence, decreased hippocampal volume in these patients cannot be considered a mere disease outcome, but may be a pre-existing risk factor and could be related to early and continued exposure to aversive situations (115).

A further example largely associated with aging is that of Alzheimer's disease (AD), in which a continuum exists comprising a preclinical stage, a symptomatic predementia stage known as mild cognitive impairment (MCI), and the final stage of dementia (116). The molecular hallmarks of MCI subjects who progress to AD show positive biomarkers of amyloid-β (Aβ) and tau-related neural injury (117, 118). Periphery biomarkers, such as lower levels of cerebrospinal fluid (CSF) Aβ, indicate increased accumulation in the brain, whereas increased CSF tau levels indicate damaged neuronal microtubules, clearly evidencing synaptic dysfunction (i.e., desynchronization and hypersynchronization) due to AD (119) that negatively impacts synaptic plasticity and causes synaptic loss, which in turn leads to impairment of neural networks involved in memory and cognition.

Studying network organization patterns in such conditions offers the advantage of addressing developmental trajectories, with causal and longitudinal interpretation and offering the possibility to differentiate primary brain circuits alterations from secondary wide-spread function loss and to highlight the involvement of particular brain regions as network nodes or connectivity dynamics as active processes (120). In this sense, network compensation as an adaptive mechanism for resilience is not only of use to explain stress coping and to closely track and predict preserved mental health (7), but can be also illustratively studied to monitor adaptation and compensation that occur during the lifespan (69, 121). While age-related decreased connections from and to the frontal regions and increased connections to the posterior (parietal) modules have been described (122) these have been closely related with maintained cognitive function (123, 124). Moreover, variations in network efficiency predict memory function across different life periods and are suggested to reflect differences in information processing between association and sensorimotor systems (125). This all points to the fact that the adjustment of network organization is responsible for buffering alterations in cognitive ability (126, 127), and the loss of optimal topological organization is associated with disease development and impaired brain function (128, 129). Hence, the capacity to react to threats is built into specific brain circuits whose development is influenced by multiple experiences and present differential susceptibility during the lifespan.

## Brain networks fingerprints of reorganization during lifespan and resilience

The dynamics of brain reorganization during lifespan partly mirror structural (130) and functional networks behavior (131, 132), increasing efficiency during maturation through young adulthood until reaching a peak at about 30–40 years of age, that acts as an inflection point, after which the efficiency of brain circuits starts to decrease and physiological aging begins. More broadly, the structural integrity of the brain, as measured by the brain parenchymal fraction (BPF) presents a similar trend across the lifespan (133), whereas the cognitive ability increases throughout young adulthood and decreases in older adulthood (134). Variations in the trajectories are expected in relation to biological and environmental factors, such as genetics, lifestyle, education, socio-cultural background, exercise engagements and learning.

**Figure 2:**
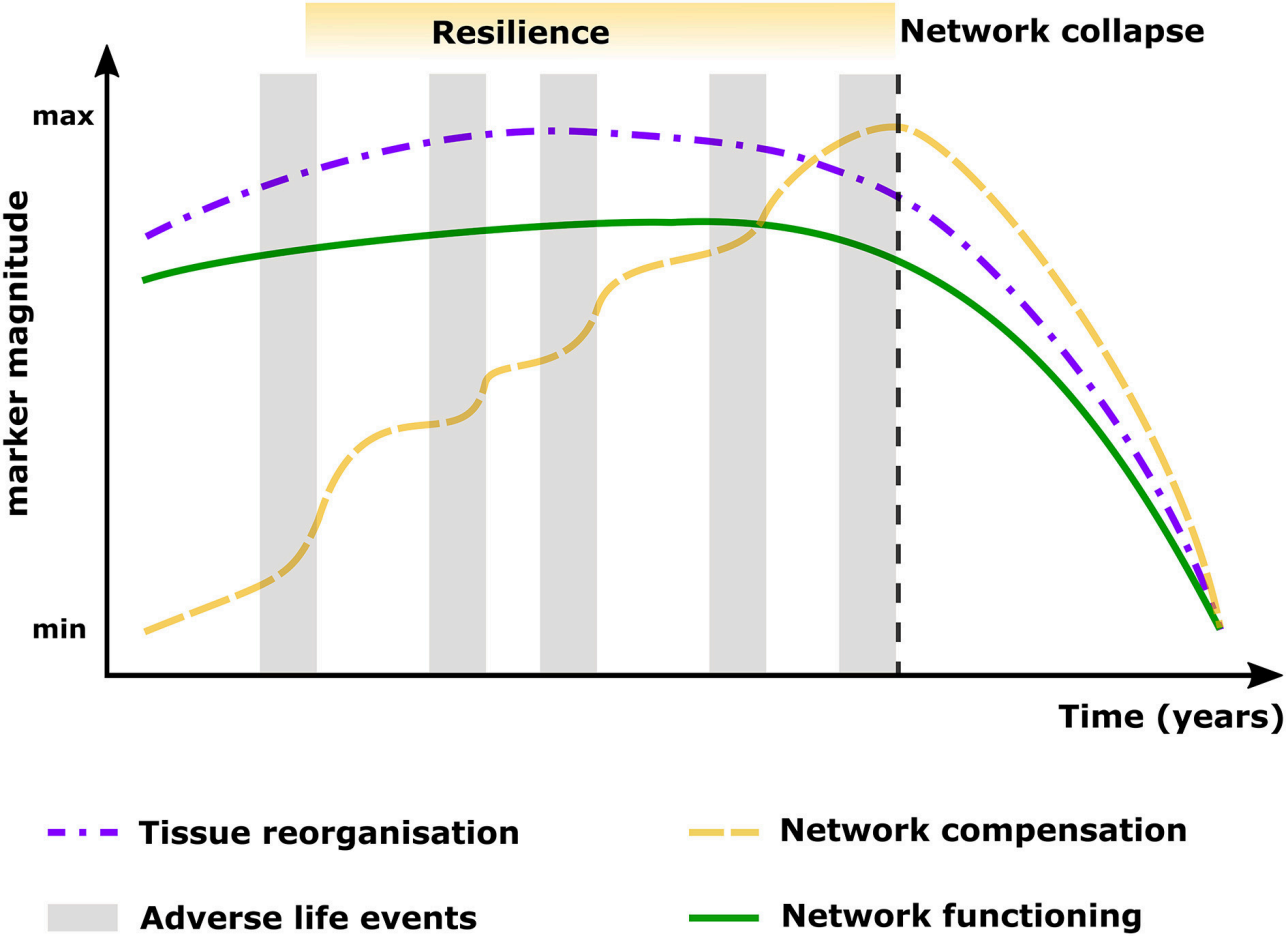
Model showing the likely developmental trends of resilience, showing the maintenance or recovery of mental health during and after exposure to significant adverse event results from a dynamic process of adaptation to the given life circumstances (gray boxes), where global reorganization (purple line) and mechanisms of compensation (yellow line) across the whole brain network are in charge of maintaining optimal functioning and efficiency (green line) in relation to cognitive ability and mental health. However, increased or sustained exposure to adverse life events or inadequate network reorganization will lead to exhaustion or collapse of the network (black dashed line), which manifests as divergent lifespan patterns or breakdown of functional responses leading to loss of mental health.

Despite the above mentioned age-related lifespan brain reorganization processes that explain links from structure to cognitive and mental function (1, 2), whether all these changes result from modifications in earlier or later life periods remains an open question. More importantly, which compensation patterns occur at different life points and how brain circuits can compensate for detrimental events is only partly understood (135–137). This is a central point of resilience research, which postulates that active processes of brain reorganization play an essential role for preserved mental function (11), where the connectivity patterns shaping excitability regulation mechanisms are principally involved in sustaining mental health and physiological trajectories of cognitive function (138). Accordingly, we propose an integrative model as shown in Figure 2.

To confront the increased endogenous challenges (i.e., changes to neural anatomy and physiology), as well as exogenous challenges (i.e., those brought about by traumatic events or by changes to the environment), brain circuits must present tremendous abilities to flexibly adapt. Maintained mental health can be generally achieved by the integrated communication between frontal and parietal brain regions (3, 4). Within this view, reorganization principles of the brain's topological architecture, beyond being mere compensatory mechanisms (47, 75), could be thought of as manifestations of neural plasticity, synaptic adaptation and reorganization of information flows (69, 139–142). This hypothesis is based on the fact that age-related functional and structural reorganization processes spatially correspond to evolving patterns of activation, and that these structure-function patterns are tightly associated with cognitive performance (1, 143). More supporting evidence shows that adults who do not show age-related adjustment in EEG theta-alpha power are more likely to exhibit cognitive deficits than those who adapt (62). Accordingly, the process of resilience cannot be restricted to early life but should operate throughout the entire lifespan.

Recent studies have emphasized that the integral topological architecture of the brain networks, i.e., modular organization, supports increased cognitive demands (144–146) and that the reorganization of its patterns are tightly related to aging (122, 125, 147). How this process of increased modularization of brain networks is related to mental health or resilience is not yet clear but should be a matter of future studies. Hence, adaptation of interregional connectivity and specific changes in network topology are likely to be contributing mechanisms to healthy aging (147, 148). This phenomenon is characterized by modification within different brain subsystems (122), which contain regions critical for several cognitive functions and/or are particularly sensitive to disease development. Specifically, the prefrontal, anterior and posterior cingulate cortices, as well as the precuneus and the inferior parietal lobe have been consistently shown to present age-related changes in connectivity relevant to preserved mental function and resilience (3, 4).

Common patterns of functional and structural network reorganization have been reported at different time scales (149), mainly involving reduced connectivity in the fronto-parietal or default mode networks which seem to be the normal adaptation to healthy aging. On the contrary, although the temporal cortex shows a similar trajectory with age as other regions (150, 151) the changes to this region show signs of neurodegeneration (152, 153). These findings pinpoint the possibility of specific regional contributions to differentiate healthy from abnormal aging trajectories, and opens the possibility that failures in age-related network reorganization predispose the brain to the development of the so-called disconnection syndromes.

In the same line, specific network topology patterns have already been related to the disease course and clinical progression in AD (154) and Parkinson's disease (16), multiple sclerosis (6, 15), schizophrenia (155, 156), depression (157) and PTSD (158, 159). These observations emphasize that the network adaptations are not merely a consequence of pathological alterations, but should be seen as integrative processes of functional and structural alterations and compensation for optimal network functioning (123), which act until the set-point at which the network performance cannot be maintained and compensation abates (7). Furthermore, patterns of network reorganization are linked to plasticity in the normal brain (160) and with maintenance of function despite continuous damage (161, 162). This suggests that the correct adaptation of distinct brain circuits may be the key mechanism underlying resilience (as in our proposed model shown in Figure 2), where parallel processes of lifespan-related reorganization in brain circuits can be drawn, and used to improve our understanding, under an holistic framework, of the interrelation between physiological and pathological developments and experienced life events (7).

## Conclusion

Clearly, brain networks development and reorganization across the human lifespan are active and continuous processes, which allow the emergence of resilience mechanisms across the entire lifespan. Within these networks, the frontal and parietal regions are certainly modulators of maintained health, where the intrinsic dynamics and excitability patterns of these regions and their connections, for instance to the temporal lobe and hippocampus, are able to reduce vulnerability and risk for damage. Hence, correct adaptation of these connectivity patterns could be the key to healthy aging and diminish the risk for developing neurological and neuropsychiatric disease at different stages across the lifespan. Future research should aim for multivariate full-brain investigations using not only larger study populations, also with the help of combined biomarkers at the micro-level, such as blood markers or genetic profiling, and at macro-level, e.g., brain imaging and network analyses; all together with longitudinal designs in order to better capture the full dynamics of lifespan modifications.

## Author contributions

GG-E, MM, JV, and SG made substantial contributions to the conception and design of the work; performed the literature search, drafted parts of the manuscript and revised it critically. GG-E, and MM prepared the first version and revised subsequent versions of the manuscript. JV and SG made substantial contributions to the interpretation of data. VC made substantial contributions to the interpretation of data; he made the figures and revised the manuscript critically. GG-E, MM, VC, JV, and SG give their final approval of the version to be published and agree to be responsible for all aspects of the work so that questions about the accuracy or integrity of any part of the work are adequately investigated and solved.

### Conflict of interest statement

The authors declare that the research was conducted in the absence of any commercial or financial relationships that could be construed as a potential conflict of interest.
